# A gridded inventory of anthropogenic hydrogen emissions in Europe

**DOI:** 10.1016/j.isci.2025.114095

**Published:** 2025-11-17

**Authors:** Marya el Malki, Antoon Visschedijk, Ingrid Super, Jesse Duroha, Anthony J. Marchese, Hugo Denier van der Gon

**Affiliations:** 1 Air quality and Emissions Research (AER), TNO, Utrecht, the Netherlands; 2 College of Engineering, University of Rhode Island, Kingston, RI 02881, USA

**Keywords:** Environmental science, Environmental resource, Environmental assessment

## Abstract

Hydrogen (H_2_) is increasingly perceived as critical to the transition to a low-carbon economy, particularly in Europe, where ambitious climate targets require drastic reductions in greenhouse gas emissions. However, hydrogen emissions are not included in current reporting obligations, and their environmental impact remains underexplored. This study provides a first gridded inventory of anthropogenic hydrogen emissions across Europe, combining bottom-up estimates with spatially resolved activity data. In 2022, emissions were about 327 ktH_2_/year (276–378 ktH_2_/year). The largest sources are internal combustion engines in road transport (∼38%) and residential wood combustion (∼35%). By contrast, emissions from the hydrogen value chain are smaller, around 83 ktH_2_/year (32–133 ktH_2_/year). However, hydrogen production is expected to increase 5- to 6-fold by 2050. Under such a scenario, hydrogen emissions from the hydrogen value chain can dominate hydrogen emissions in Europe and could undermine the climate benefits of hydrogen as an energy carrier, if not properly managed.

## Highlights

• Europe emitted ∼327 kt of hydrogen in 2022 from anthropogenic sources
• Conventional combustion sources dominate: road transport and wood burning
• Hydrogen value chain losses were ∼83 kt/yr, could dominate with hydrogen scale-up
• High-resolution maps reveal emission hotspots for monitoring

## Graphical abstract

**Figure:**
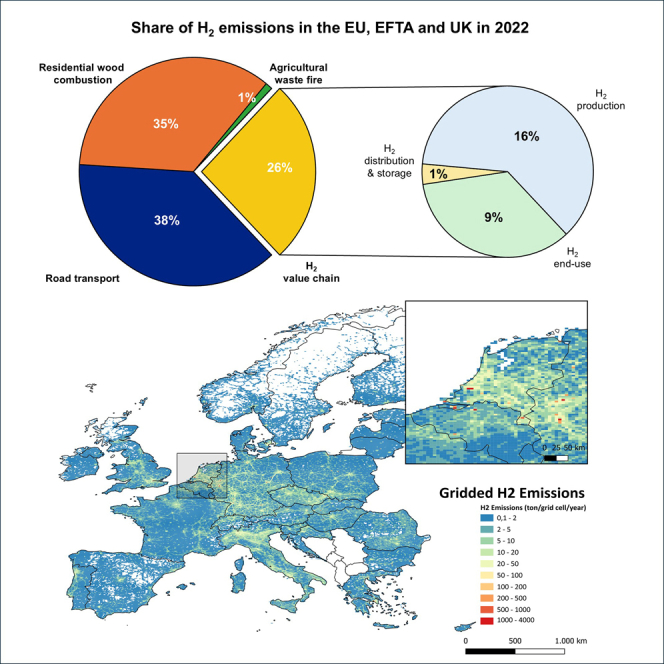

## Introduction

It is essential to develop and use low carbon energy sources to avert the worst impacts of climate change.^1^ Hydrogen (H_2_) is gaining attention as a clean fuel supporting a transition to a decarbonized energy system. As such, it features in all the European Commission’s future net zero emissions scenarios (EC, 2018), and European governments are increasing their efforts to boost hydrogen technologies, infrastructure, and applications. However, several recent studies have highlighted the possible impact of changes in the atmospheric hydrogen concentration that might arise from emissions from all stages in the production, distribution, storage, and utilization of hydrogen.^2^^,^^3^ Hydrogen has indirect warming effects on the planet due to its chemical reactions with other atmospheric compounds. When hydrogen is released into the atmosphere, the bulk (70–80%) is eventually oxidized by (soil) bacteria, but around 20%–30% is oxidized by reacting with the naturally occurring hydroxyl radical (OH).^4^^,^^5^^,^^6^ This process contributes to global warming as it leads to less OH available for methane oxidation, thereby prolonging the lifetime of this potent greenhouse gas. Moreover, the follow-on reactions of hydrogen with OH ultimately lead to the formation of tropospheric ozone and stratospheric water vapor,^2^ which also contributes to global warming.^7^

The climate impact of hydrogen, therefore, depends strongly on the extent of emissions along the value chain. Recent studies have started to quantify these emissions directly,^8^^,^^9^ but large uncertainties remain regarding their magnitude, variability, and distribution across technologies and regions. To fully grasp the potential climate consequences of scaling up hydrogen technologies, it is essential to gather robust data on hydrogen emissions throughout the value chain, including the localization of production, storage, transportation, and end-use. This highlights the need for a spatially resolved and consistent inventory of hydrogen emissions, which is essential for chemical transport modeling. Such an inventory will help identify potential hotspots and key sources of hydrogen emissions, crucial for a dedicated monitoring strategy, and support a framework that could minimize the climate impacts of a future hydrogen economy.

In this study, we focus entirely on anthropogenic hydrogen emissions. These emissions, mainly from fossil fuel use and biomass burning, are estimated at about 25,000–30,000 kton H_2_/year. While smaller than the combined biogenic sources, such as marine or soil emissions or the oxidation of volatile organic compounds, they still represent a substantial share of the global hydrogen budget.^10^^,^^11^ The full environmental impact of hydrogen use, however, also relies on its production method, including associated CO_2_ and/or CH_4_ emissions. While we will not assess associated emissions, we refer to different production methods following the commonly used colors: “gray” hydrogen made using natural gas, “blue” hydrogen referring to gray hydrogen with additional Carbon Capture and Storage (CCS), and “green” hydrogen produced from water by electrolysis using renewable energy sources. For an overview of the entire so-called hydrogen rainbow, we refer to Rodríguez (2022).

This study sets out to compile a spatially resolved inventory of hydrogen emissions in the EU27, EFTA countries, and the UK, for the year 2022. The emission sources considered are the direct losses of pure or mixed hydrogen (syngas only) to the atmosphere, resulting from production, transport, and end-use (consumption) of hydrogen, and hydrogen formed in combustion reactions. Besides estimating how much hydrogen is emitted, this inventory specifies where, in a geographical sense, these hydrogen emissions occur. The main objective of the study is an emission database specifying hydrogen emission from point sources at exact location (if possible) and area sources at a resolution of ∼6 × 6 km, as used in the standard Copernicus Atmospheric Monitoring Service European emission datasets.^12^

### Background

#### Hydrogen value chain in Europe

The European Hydrogen Observatory (EHO) provides a detailed inventory of the hydrogen landscape in Europe in 2022 in its Hydrogen Market Landscape report.^13^ In Europe, hydrogen’s current primary use is as an industrial chemical to produce other chemical compounds and fuels. According to EHO (2024a), the total production of hydrogen in 2022 in the EU27, European Free Trade Association (EFTA) countries, and the UK amounted to 8,200 kton, of which 57% is used in oil refineries, 24% used to produce ammonia, 3% is used to produce methanol, and 9% to produce other chemicals. Of the remaining 7%, 3.4% is directly used as an industrial fuel, whereas only 0.1% was used for other emerging applications as an energy carrier (e.g., automotive fuel). The use of the remaining 3.5% is unknown.

Total European production capacity was 11,300 kton in 2022.^14^ The bulk of this capacity (87%) is located directly onsite where it is consumed (captive hydrogen production), while the remaining 13% is produced for external distribution and sale (merchant hydrogen production), mostly through pipelines. To produce hydrogen, there are three widely recognized pathways. Currently, the most important one is through the chemical conversion of methane (steam methane reforming, SMR), accounting for 91.2% of all hydrogen produced in Europe.^14^ Another 8.6% is produced as a chemical by-product, primarily in the production of chlorine, chlorate, ethylene, and styrene. The third pathway is by water electrolysis. In 2022, only 0.3% of the European hydrogen was produced by water electrolysis^14^ but water electrolysis is becoming more popular, while steam reforming is on the decline as a result of structurally high natural gas prices.

#### Non-combustion hydrogen emissions

Everywhere hydrogen is handled, losses to the atmosphere can occur, and being such a small molecule, hydrogen is prone to leakage.^15^ Losses may be the result of direct equipment and pipeline leakage, venting and purging of hydrogen-containing waste or process streams, diffusion/permeation through materials, boil-off from liquid storage, and other (un)intentional releases of hydrogen. The more hydrogen is handled, the higher the potential losses may be. This study, therefore, focuses on the industrial usage of hydrogen, although emerging use as an energy carrier will be considered too.

As hydrogen has not received much attention as a climate pollutant before, validated emission estimation methods, including emission factors or loss rates, have not yet been developed. There have nonetheless been attempts to quantify hydrogen emissions from its value chain, or parts of it. Esquivel-Elizondo et al. (2023) summarize the results of an extensive literature search on hydrogen loss fractions. They made indicative estimates of the range in which real-world loss fractions of hydrogen may reside, but noted that their work was hampered by a severe lack of experimental data.

#### Combustion hydrogen emissions

Hydrogen emissions from the industrial handling of hydrogen are not the only significant anthropogenic source of hydrogen emissions. Popa et al. (2015) highlighted gasoline-fueled vehicles as a significant contributor, drawing on earlier findings by Bond et al. (2010) that identified a correlation between hydrogen and carbon monoxide (CO) in vehicular exhaust. Although direct measurements of exhaust gas composition, including hydrogen, remain limited, subsequent experimental studies have confirmed the presence of hydrogen in vehicle emissions.^16^^,^^17^

Biomass combustion and open burning have also been identified as important sources of anthropogenic hydrogen. Vollmer et al. (2012), using both literature data and measurements from residential wood combustion, observed relatively consistent hydrogen-to-CO ratios across different types of wood burning and plant material combustion. On a global scale, they estimated that 40–50% of anthropogenic hydrogen emissions may originate from these processes.

## Results

The contributions of various hydrogen sources and/or uses to hydrogen emission across Europe are calculated as described in the STAR Methods section, resulting in a first spatially distributed European hydrogen emission inventory. The inventory is supplied as two data products: the inventory, which is a database, and the gridded maps where the emissions are grouped by sectors and spatially distributed using TNO’s spatial proxies as described in Kuenen et al. (2022).

The results presented in this section represent the hydrogen emissions based on the mean value for emission rates per source. The corresponding ranges of emissions, provided in Tables S2 (production), S3 (by-product production), S4 (end-use), and S5 (refueling stations), reflect the uncertainty ranges in emission rates reported by Esquivel-Elizondo et al. (2023).

### Major contributors to hydrogen emissions

**Figure 1:**
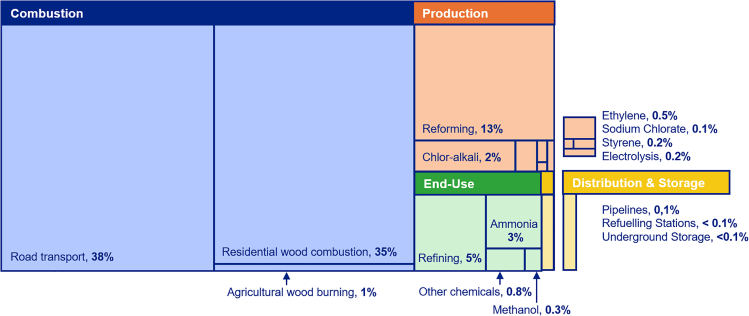
Share of hydrogen emissions (%) from different sources in the hydrogen value chain and combustion sources, based on mean hydrogen emission rate values

The contribution of every source to the total hydrogen emissions in the selected European domain (EU27, EFTA, and UK) is illustrated in Figure 1. Internal combustion engines used in road transport and the residential combustion of wood are the two dominant sources, accounting for respectively 38% and 35% of total hydrogen emissions in 2022.

Table 1 focuses specifically on the non-combustion emissions, detailing the share (%) of hydrogen emissions from the hydrogen value chain alone. Hydrogen emission within the hydrogen value chain is primarily driven by gray hydrogen production with methods such as SMR and the end-use of hydrogen in large-scale industrial applications such as oil and gas refining and ammonia production. Although these processes generally have lower emission rates, as seen in Table 2, their large operational volumes result in significant emissions. In contrast, electrolysis currently makes a smaller contribution due to the limited scale of green hydrogen production in Europe. However, as identified in the literature, electrolysis has relatively high emission rates, and with the expected expansion of renewable energy-powered hydrogen production, emissions from electrolysis could become more significant in the future. To provide a clearer understanding of hydrogen emissions, Table S1 presents the absolute values, offering a more tangible perspective on the scale of emissions from each source. A breakdown per country is depicted in Figure S4.

**Table 1:** Share of hydrogen emissions (%) from the hydrogen value chain (excluding the combustion of fuels)

Process	Sub-process	Share of Hydrogen Emissions (%) from the value chain
Production	Electrolysis	1%
Production	SMR with and without CCS	50%
Production	By-Product	13%
Pipelines	–	3%
Refueling Stations	–	< 0.1%
Underground Storage	–	< 0.1%
End-Use	Ammonia	10%
End-Use	Methanol	1%
End-Use	Other Chemicals	3%
End-Use	Refining	19%

**Table 2:** Emission rates summary table (based on the review by Esquivel-Elizondo et al. (2023))

Component	Source	Mean	Min	Max
Production	Gray and Blue H_2_	0.55%	0.1%	1%
Production	Green H_2_	3%	2%	4%
Transportation	Transportation & Storage Pipeline Leaks	67.7 kg/km	–	–
Transportation	Mass H_2_/Mass CH4	0.35	–	–
Distribution	H_2_ Gas Refueling Stations	2%	0.25%	3%
Underground Storage	Salt Cavern	0.04%	0.02%	0.06%
End-Use	H_2_ End-Use	0.35%	0.2%	0.5%

**Figure 2:**
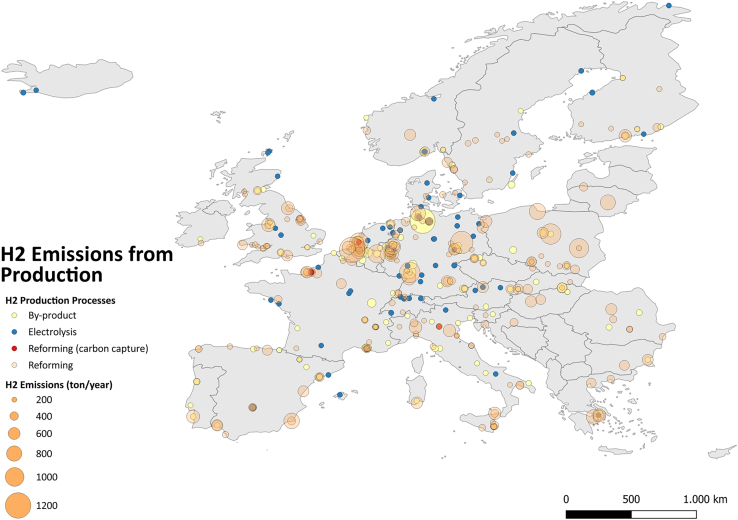
Hydrogen emissions from different production processes across the European domain (EU27, EFTA, and UK) in 2022

Figure 2 shows that reforming and by-product hydrogen production are a significant source of hydrogen emissions across Europe, particularly in industrial clusters such as around Rotterdam, Antwerp, and in North Rhine Westphalia, where large-scale hydrogen production facilities are concentrated. These regions house many large SMR plants, mainly producing gray hydrogen without CCS technologies.

As previously mentioned, while gray hydrogen has a lower emission rate on average compared to green hydrogen, its operational capacity is much larger due to the higher number of installed plants and their increased utilization. This underscores the continued reliance on conventional hydrogen production methods in Europe, particularly in regions with well-established fossil fuel industries.

### Hydrogen emissions from pipelines, refueling stations, and storage

#### Pipelines

At present, Europe’s hydrogen pipeline network remains limited, and its contribution to overall emissions is moderate, as seen in Table 1. However, with the anticipated expansion of the hydrogen economy, pipeline networks are likely to grow, necessitating further efforts to reduce emissions across these distribution systems. Hydrogen emissions from pipeline networks are currently concentrated in Western Europe, where most of the existing hydrogen infrastructure is located (e.g., the Air Liquide networks in Benelux, Germany-and-France). Our analysis is based on the total length of pipelines. This method could be refined in the future to include additional factors, such as throughput, pressure levels, and the presence of compressor units, which are known to affect emission rates, provided such data become available. Additionally, truck deliveries that supply these pipelines may also contribute to overall emissions and should be included in future assessments.

#### Refueling stations

Refueling stations currently account for a very small portion of hydrogen emissions (Table 1). Our study focused primarily on gaseous hydrogen refueling stations, as liquid hydrogen refueling remains less prevalent. Given the small fleet of hydrogen-powered vehicles currently in operation, emission rates from refueling infrastructure are relatively low. However, this may change as hydrogen vehicles become more widespread and refueling networks expand. The current state of refueling station infrastructure, shown in Figure S3, provides further insights into the current spatial distribution of this growing sector.

#### Underground storage

Hydrogen storage, while crucial for balancing supply and demand in the future hydrogen economy, currently plays a limited role in emissions (Table 1). Our study identified only one operational salt cavern used for hydrogen storage, contributing minimally to overall emissions. As hydrogen production and usage scale up, additional storage solutions will likely be developed, and their potential emissions will need to be addressed.

### Hydrogen from end-use sectors

**Figure 3:**
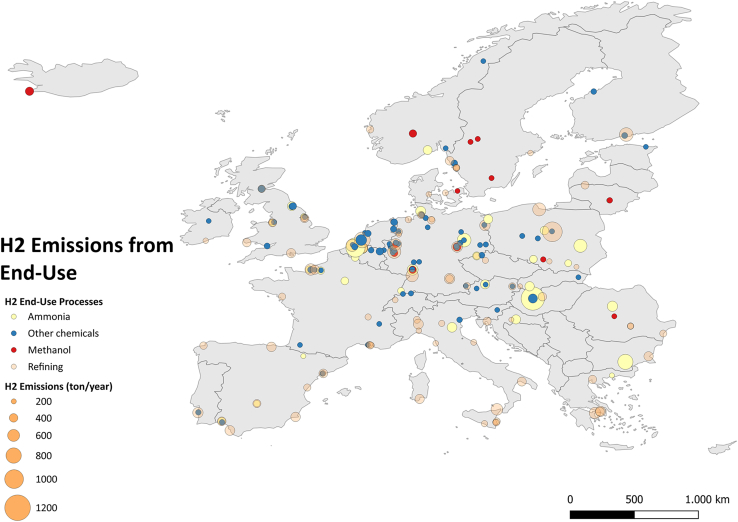
Hydrogen emissions from different end-use processes across the European domain (EU27, EFTA, and UK)

Figure 3 depicts hydrogen emissions from various end-use processes across the European domain, with notable clusters of activity identified in regions such as Rotterdam, which houses significant refining and chemical production facilities. These sectors are the most prominent contributors to hydrogen emissions from end-use, especially in industrial processes such as ammonia and methanol production and other chemical industries. Because a uniform emission rate was used for all end-uses due to data scarcity (as described in the STAR Methods section), comparing the contributions of different chemical manufacturing types beyond their capacity remains challenging.

Our findings also highlight the presence of other industrial clusters, though the picture is evolving rapidly. Even though this is currently not reflected in the inventory, many ammonia and methanol plants have reduced operations or shut down indefinitely due to high gas prices, market, and/or geopolitical pressures, such as the war in Ukraine.^18^ This has led to a temporary reduction in ammonia-related hydrogen emissions, but this may change as economic conditions stabilize.

Other end-use applications, such as fuel cells, currently play a minor role in hydrogen emissions, but they may become more relevant in the future as hydrogen adoption expands across sectors such as transportation and power generation. Future assessments should account for emerging end-use technologies that may alter the distribution of hydrogen emissions across Europe.

### Hydrogen from combustion

As mentioned previously, road transport currently appears to be the dominant source of hydrogen emissions, resulting in a distribution pattern that has a substantial influence on the distribution of total hydrogen emissions. Larger population centers, such as cities, and the road network are clearly visible, highlighting that hydrogen is emitted not only from specific point sources, but also diffusely across the road network. The spatial distribution of combustion emissions of hydrogen in road transport is shown in Figure S5.

A second major source of anthropogenic hydrogen emissions is the residential combustion of biomass, particularly fuelwood. The relative contribution of this source is estimated to be comparable to that of road transport. However, its spatial distribution is less distinct: while it broadly follows population density, it is more strongly associated with rural, forested areas than with urban centers. This distribution pattern is illustrated in Figure S6.

### Gridded hydrogen emissions in Europe

**Figure 4:**
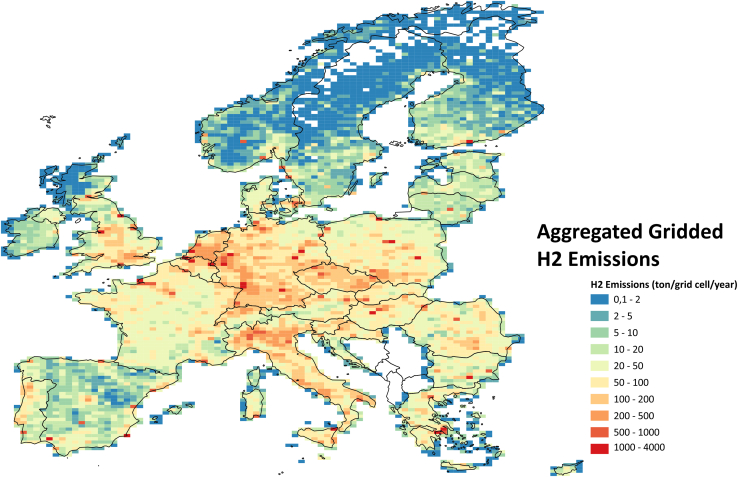
Aggregated hydrogen emissions in 2022 (in ton/grid cell/year) from all sources across the domain, at a coarser resolution of 0.5° × 0.25°

Figure 4 shows the gridded hydrogen emissions for the whole domain for all sources and how they are distributed over Europe. To improve the visibility of individual large sources, the resolution of the grid in the figure has been reduced from 0.05 ° × 0.1 °–0.5° × 0.25° latitude longitude. Note that this is only for presentation purposes; the emission dataset prepared in this study is made for the EU27, EFTA countries, and the UK at a high resolution of 0.05 ° × 0.1 °. Hydrogen emission is concentrated in a densely populated region in and around the Netherlands and the UK, with lots of industrial activity and traffic.

**Figure 5:**
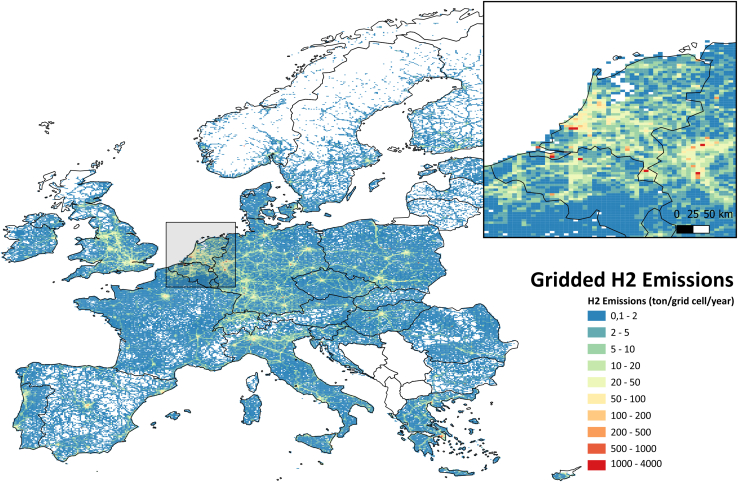
Hydrogen emissions in 2022 (in ton/grid cell/year) from all sources across the domain, including a closer look at Antwerp, Rotterdam, and North Rhine-Westphalia (near Düsseldorf) at a resolution of 0.05° × 0.1°

To understand the patterns better in this region, Figure 5 shows the gridded total hydrogen emission across the domain, with a zoom in on Northwestern Europe at a resolution of 0.05 ° × 0.1 °. The figure illustrates the gradients present in the data. Point sources (mostly emissions from large SMR plants) are clearly visible in the plot as small distinct red squares (>1000 ton H_2_/cell/year), while areas with a lot of road traffic such as the Dutch Randstad region (including Rotterdam, Amsterdam) and German North Rhine Westphalia region (including Dusseldorf, Essen, Duisburg) show up as bigger orange colored zones (with 50 to several hundreds of ton H_2_/cell/year). Some busy highways are recognizable as longer stretches of green parts with cell totals of 10-to-100-ton H_2_/cell/year. Emissions from residential fuelwood use appear less distinct, since this source has a more diffuse spatial distribution. Depending on the distance to source and wind direction, local ambient concentrations of hydrogen may be primarily determined by either road transport and/or residential biofuel use, or when close, industrial hydrogen production or use.

## Discussion

The transition to a hydrogen-based energy system offers substantial potential for reducing Europe’s carbon emissions, but several studies (e.g., Ocko & Hamburg (2022) and Warwick et al. (2022)) highlight the need to address hydrogen emissions to ensure its environmental benefits. In this study, we have developed a first gridded hydrogen emission inventory for Europe, a critical component for a comprehensive assessment of the environmental benefits of a (future) hydrogen economy.

One key finding from our work is the current dominance of hydrogen emissions from combustion processes (internal combustion engines and residential wood combustion in particular), contributing nearly 77% of total anthropogenic emissions. This underlines the continued significance of combustion-related hydrogen emissions. In this study, we have focused on anthropogenic sources of hydrogen. While biomass burning from residential wood combustion and agricultural waste burning are included, sources such as wildfires were out of scope as it semi-natural. We have also not incorporated industry and power sector emissions. Vollmer et al. (2012) suggest that these are generally minor compared to transport and residential wood combustion, with the possible exception of hydrogen emissions from residential coal combustion. The latter could be relevant but remains poorly quantified and could therefore be considered in future work as part of a broader effort to include additional fossil fuel and biomass combustion sources in assessments of anthropogenic hydrogen release. Recognizing and quantifying the contribution of combustion is particularly important when establishing a baseline for future measurements and monitoring efforts.

Within the hydrogen value chain, hydrogen loss is primarily dominated by SMR production, which is concentrated in industrial areas such as the harbor areas of, for instance, Rotterdam, Antwerp, as well as the North Rhine-Westphalia industrial region. End-uses such as ammonia production and refining, also located in these regions, further contribute to hydrogen losses, making these industrial clusters key areas for further exploration and potential measurement campaigns. Hydrogen storage and transport are at the moment of limited importance because the bulk of the hydrogen produced is currently also consumed at the same (industrial) location. Green hydrogen through electrolysis currently has a very limited capacity in Europe, making it less significant for emissions today, though it may become more relevant in the future as the hydrogen economy grows. Expectations are that hydrogen production will increase 5- to 6-fold from around 8,000 kton/year to 42,000 kton/year in 2050, with an upper limit of 63,000 kton/year (EHO, 2024e). Under such a scenario, hydrogen emissions from the hydrogen value chain can dominate the total hydrogen emissions in Europe. Moreover, if taking place at remote locations, large-scale electrolysis may bring about a need for large-scale liquification for hydrogen transport and storage purposes, including the emissions that this would entail.

The gridded hydrogen emission data can be used in atmospheric chemistry modeling studies. Moreover, the emissions per unit of activity can be used in scenario studies to estimate future emissions under various development pathways. An example worth considering in this respect would be the effect on the total hydrogen emission of a large-scale introduction of hydrogen fuel cell electric vehicles (FCEVs) in combination with a phase-out of combustion engines in road transport.

The accuracy of this inventory is subject to certain limitations. The lack of extensive observational data and reliable emission factor measurements for all hydrogen emitting activities introduces significant uncertainty into the emission estimates. Combustion emissions are identified as an important source. Our current estimation of combustion-related hydrogen emissions is based on exhaust/flue gas measurements of H_2_ and CO reported by Bond et al. (2010, 2011) and Vollmer et al. (2012), in agreement with other experimental studies.^17^^,^^19^^,^^20^^,^^21^^,^^22^^,^^23^ All studies consistently report a strong correlation between the two pollutants (see method details for additional information). Given that CO emissions from the current vehicle fleet are much better known, we apply measured H_2_/CO ratios to estimate total H_2_ emissions from road transport. Thus, the detailed breakdown of the vehicle fleet, such as variations by vehicle types and Euro emission standards, mirrors the variation in CO emissions, with distinct H_2_/CO ratios applied to older versus newer Euro classes as well as to two- and four-wheeled vehicles. Further disaggregation of H_2_ emissions by operating mode (e.g., cold start conditions) will be important for refining the inventory and capturing emissions not directly tied to hydrogen production but still contributing to overall hydrogen release. Regarding residential wood combustion, Vollmer et al. (2012) measured H_2_/CO ratios for different types of wood combustors varying by a factor of five, suggesting a significant influence of appliance and biofuel type. They, however, reported a much lower overall uncertainty in the average H_2_/CO ratio for residential biofuel combustion (±20%) in their conclusions. A similar range may be tentatively adopted for our hydrogen emission estimate from this source, but more research would be needed here.

Another uncertainty is the role of intentional purging and/or venting of hydrogen, which may happen especially during green hydrogen production during start-up and shutdown. Currently, this is a minor source as green hydrogen production is still very limited. In the future, facility scale monitoring, e.g., by **collecting** downwind measurements over longer periods, would be needed to have a better quantification of this process. Future research should also aim to refine our emission estimates by collecting more observation-based emission rates and building a broader experimental dataset to better quantify uncertainties in emission factors. New measurements may lead to expanding the inventory by including more detail on sources outside of the H_2_ value chain, such as other conventional activities potentially emitting hydrogen-containing gases (e.g., iron and steel production, coke ovens). Furthermore, new measurements from upcoming activities related to the hydrogen value chain such as hydrogen transformation processes, above-ground liquid hydrogen storage, and emissions from fuel cell electric vehicles, will lead to better quantified emissions. All the above identified knowledge gaps support the need for more measurements to further improve our present-day baseline of hydrogen emissions.

In conclusion, while hydrogen holds great promise for decarbonization, addressing its potential environmental impacts, particularly its emissions, is crucial for realizing a truly sustainable energy transition. As illustrated by Ocko and Hamburg (2022), the climate change mitigation potential of clean hydrogen alternatives is determined by the associated hydrogen emission rates, with substantial differences between the climate benefits or disbenefits from lower-end emissions (1%) compared to higher-end emission rates (10%). The present inventory is a major step forward toward our ability to make integrated assessments of the environmental impacts of decarbonization pathways with or without hydrogen, identifying current key sources for effective monitoring strategies and informing policy-making. Regardless, minimizing hydrogen emissions at all stages of the value chain is a no-regret policy and is highly recommended.

### Limitations of the study

This study provides a first high-resolution gridded inventory of anthropogenic hydrogen emissions in Europe. However, several limitations remain. For the hydrogen value chain, emission factors are largely based on literature-derived values, and uncertainty is still considerable due to the lack of direct, systematic measurements. Future measurement campaigns at facilities and infrastructure sites will be essential to better constrain loss rates across production, transport, storage, and end-use. For combustion sources, our estimates rely on applying H_2_/CO ratios from previous studies to known CO emissions. While this approach is consistent with available experimental evidence, it introduces uncertainty in absolute emission levels and may not fully capture variability across appliance types, vehicle categories, or operating conditions. Another potential caveat could be hydrogen emissions from anthropogenic processes that involve methanogenesis, such as composting, landfills, and biogas production. Currently, measurements and derived emission factors for these processes are lacking.

The inventory also does not yet include all potential emission sources along the hydrogen value chain. Limited information on capacity or geolocation meant that, for example, fuel cell electric vehicles, compressor stations, and above-ground storage were not represented. Although these sources are currently minor, they may become more relevant as hydrogen deployment expands. More broadly, the focus on anthropogenic sources excludes (semi-)natural contributions such as soils or wildfires, as well as some industrial activities where data are lacking. While these limitations should be kept in mind, the inventory provides an important baseline for Europe and identifies clear priorities for improving data and refining estimates in the future.^24^^,^^25^^,^^26^^,^^27^^,^^28^^,^^29^^,^^30^^,^^31^^,^^32^^,^^33^^,^^34^^,^^35^^,^^36^

## Resource availability

### Lead contact

Requests for further information and resources should be directed to and will be fulfilled by the lead contact, Marya el Malki (marya.elmalki@tno.nl).

### Materials availability

This article does not generate new unique materials.

### Data and code availability

• Country-aggregated and plant-by-plant data are available in the supplemental information section.
• Gridded emission maps for 2022 for the European domain are available from the lead contact upon request.
• Any additional information required to reanalyze the data reported in this article is available from the lead contact upon request.

## Acknowledgments

The research leading to these results has received funding from the 10.13039/100020074Environmental Defense Fund.

## Author contributions

Conceptualization, M.M., A.V., and H.D.G.; methodology, M.M., A.V., I.S., J.D., and A.J.M.; investigation, M.M. and A.V.; writing – original draft, M.M., A.V., and H.D.G.; writing – review and editing, M.M., A.V., H.D.G., and I.S.; funding acquisition, H.D.G.; supervision, H.D.G.

## Declaration of interests

The authors declare no competing interests.

## STAR★Methods

### Key resources table

**Table:** 

REAGENT or RESOURCE	SOURCE	IDENTIFIER
**Deposited data**		

CO spatial distribution grids for road transport emissions, residential biomass and agricultural waste burning	CAMS-REG-v4	https://doi.org/10.24380/0vzb-a387
CO emission data for road transport emissions, residential biomass and agricultural waste burning for 2022	CAMS-REG-v8.1	https://eccad.sedoo.fr/#/metadata/608
Hydrogen production capacity and demand	European Hydrogen Observatory	https://observatory.clean-hydrogen.europa.eu/tools-reports/datasets

**Software and algorithms**		

QGIS	QGIS Geographic Information System	https://www.qgis.org/

### Method details

#### Identification of anthropogenic sources of hydrogen

The first step in creating a detailed inventory of hydrogen emissions across Europe involved identifying the key hydrogen streams. The primary data source for hydrogen across the value chain in Europe was the European Hydrogen Observatory (EHO), an industry-sponsored online data platform. The key potential sources of emissions included hydrogen production, end-use, transport, and distribution by refueling stations for FCEVs.^14^^,^^37^^,^^38^^,^^39^ The general methodology to assess atmospheric hydrogen losses followed in this study is outlined in Figure S3.

#### Hydrogen activity data collection and processing

##### Production and end-use of hydrogen

To develop a gridded inventory, it was imperative to obtain detailed geo-specific activity data for individual sources, such as plants, stations, and pipelines locations. This required gathering detailed information on the capacity and precise location of each plant. The hydrogen production processes were categorized into three main types: SMR with and without CCS, water electrolysis, and by-product production.

Data for hydrogen production through SMR were sourced from EHO (2024a), supplemented with data for refineries by Concawe (2024), for ammonia production by Dowling et al. (2022), and for merchant data Hydrogen Analysis Resource Center (2015). To address data gaps, we cross-referenced government reports, news articles on plant openings and closures, corporate websites of producers, and industry and market reports. Additionally, we identified unknown plant locations using Google Maps. A more detailed overview of the gap-filling process is presented in Figure S4.

For industrial by-product production, data was obtained from EuroChlor (2023) for chlor-alkali processes and Petrochemicals Europe (2021) for ethylene cracker processes. The EHO data specified country hydrogen by-production totals per chemical product^14^ The relative hydrogen production share of each plant was estimated based on their chlorine and ethylene production capacity share. As a validation step, the quantities of chlorine and ethylene were converted based on the stoichiometric coefficients of the reactions to ensure consistency across different sources. This revealed only minor deviations, confirming the validity of our approach. Data for sodium chlorate and styrene production was derived from various sources, including plant operator websites and Ihonen et al. (2020) for Finland, Norway, and Sweden. The aggregated capacities (aggregated by end-use per subprocess type per country) reported by EHO (2024b) were preserved, while individual plant capacities were gap-filled by scaling them according to end-use per country. In rare cases, there was no specific plant information available, and the aggregate capacity per end-use was then evenly distributed per country across the respective plants.

The primary end-uses of hydrogen include ammonia production, oil refining, methanol production, and other chemical production. Hydrogen loss from end-use has been treated as a separate potential source in this study, besides production. The location of most of these hydrogen consuming plants could be derived from the collocated captive SMR production plants discussed earlier. When unavailable from EHO, the capacity data for hydrogen consuming plants like ammonia and methanol producers were collected from other literature, including corporate websites, industrial directories, and other data platforms.

The EHO (2024a) dataset for SMR and by-product, both aggregated and detailed, was complete for only a few countries: France, the Netherlands, Poland, and Spain. For the remaining countries, gap-filling like described was necessary. Additionally, there were slight discrepancies in the total number of plants between the aggregated and individual EHO plant data, with the aggregated data showing in total five more plants that could not be identified in the detailed dataset. In such cases, the total capacity was distributed among the identified plants per country. The data presented by EHO primarily reflects the status of plants in 2021, which was preserved even though some plants, particularly ammonia plants, appear to have shut down in more recent years due to the ongoing natural gas crisis tied to the war in Ukraine. For certain plants, such as methanol and ammonia end-use facilities, the gap-filling data, including plant identification and production proportions, were sourced from older references like Zomer et al. (2020). In contrast to SMR and by-product hydrogen production, for electrolysis, minimal gap-filling was required as the EHO data were largely complete.

##### Distribution, storage and refueling stations of hydrogen

For pipelines, data was sourced from the EHO (2024c). We applied choked flow calculations, which gave a mass ratio of 0.36 between H_2_ and CH_4_ due to differences in their specific heat ratios and molecular weights.^40^ As for refueling stations, data was sourced from the EHO (2024d) and gap-filled using information from operator websites. Despite having point source data for locations and capacities, determining the number of customers utilizing these facilities posed some challenges. Data from the European Alternative Fuels Observatory^41^ on the European Alternative Fleet for hydrogen were used to calculate the distribution of different vehicle types (passenger cars, light-duty commercial vehicles, and medium- to heavy-duty vehicles) per country. Due to limited specific data, an average yearly demand for hydrogen per refueling station per country was calculated based on the following equation. Vehicles were differentiated into three categories, passenger cars, light-duty and heavy-duty vehicles and an assumed yearly demand of hydrogen equal to 120 kg/year, 175 kg/year and 6000 kg/year, respectively.^42^

$$D_{H2}=\frac{\left(M1\right)\times D_{PC}\times \left(N1\right)\times D_{LDV}+\left(\left(M2+M3\right)+\left(N2+N3\right)\right)\times D_{HDV}}{HRS}$$

Where, based on UNECE standards for vehicle classification:

• *M*1,*N*1: Numbers of passenger cars and light-duty vehicles (LDVs) per country, respectively
• *D*_*PC*_: Assumed demand of hydrogen per year per passenger car (kg/year)
• *D*_*LDV*_: Assumed demand of hydrogen per year per LDV (kg/year)
• *M*2,*M*3,*N*2*N*3: Numbers of heavy-duty vehicles (HDV) per country
• *D*_*HDV*_: Assumed demand of hydrogen per year per HDV (kg/year)
• *HRS*: Number of hydrogen refueling stations per country
• *D*_*H*2_: Average yearly demand of hydrogen per refueling station per country

For compressed hydrogen storage only underground storage was considered, due to a lack of data on existence and locations of any other form of storage. Despite there being numerous plans for future underground storage projects across Europe, such as using depleted oil and gas fields and aquifers, at present only one salt cavern operational as hydrogen storage has been identified in Teesside, UK.^43^ The usable hydrogen capacity was determined using the modelling approach described by Duroha et al. (2025). First, the ideal gas law was applied to estimate hydrogen density, based on the average operating pressure. This density was then used to calculate the usable hydrogen capacity, factoring in the reported storage volume and assuming a 50% ratio of usable hydrogen capacity to cushion gas volume.^44^ Additionally, assuming 10 injection/extraction cycles per year, the annual injection activity was estimated.^45^

Storage of liquid hydrogen has also not been addressed in this study. To avoid the high costs of liquification, production and usage of hydrogen as an intermediate chemical is usually situated in close vicinity to each other, so hydrogen can be transported from one to the other using pipelines. Reported hydrogen emission from storage tank boil-off is however highly significant (see e.g., Derking et al., 2019), if the tank is not vacuum insulated. Future large-scale use as an energy carrier may lead to a considerable increase in the need for liquid storage and distribution.

#### Literature review of hydrogen emissions estimates

To estimate hydrogen emissions rates, we use emission rates provided by Esquivel-Elizondo et al. (2023) from their literature review. Recognizing the current lack of empirical data on H_2_ emissions from infrastructure, Esquivel-Elizondo et al. synthesized existing studies to provide an overview of emissions across the hydrogen value chain. Given the limited research that addresses emission estimates for both the entire value chain and its individual components, their review primarily consolidates findings from a select group of publications,^46^^,^^47^^,^^48^^,^^49^^,^^50^ ranging from production to end-use. These estimates serve as an important, albeit preliminary, benchmark for quantifying hydrogen emissions until more precise field measurements and/or loss rates can be obtained.

Esquivel-Elizondo et al. (2023) discuss the variability in estimated hydrogen emissions, noting that these estimates are based on differing methodologies, often relying on unsubstantiated assumptions, as well as proxies, in-lab experiments, and simulations or models. The significant variations in reported emissions rates can partly be attributed to the different regional focuses of the reviewed studies. While some studies estimate current emissions, others project future emissions, resulting in a broad range of estimates across various components of the hydrogen value chain.

For production, Esquivel-Elizondo et al. (2023) reported emissions rates ranging from 0.5% to 1.0% for grey hydrogen, 0% to 1.5% for blue hydrogen and 0.03% to 9.2% for green hydrogen. Blue hydrogen potentially has higher leakage risks than grey hydrogen due to additional separation processes, whereas green hydrogen may exhibit higher hydrogen emissions compared to other methods due to the underlying electrolysis process.

The review also identified that liquid hydrogen (LH_2_) stages have the broadest range of estimated emissions with 0.15% to 10.0% for liquefaction, 2.0% to 13.2% for trucking, 2.0% to 20% for handling, and 2.0% to 15.0% for refueling stations. For gaseous hydrogen, the estimated emissions for transport, storage, and usage also varied widely, with transmission pipelines ranging from 0.02% to 5.0%, distribution pipelines from 0.0003% to 5.0%, above-ground storage from 2.8% to 6.5% and gaseous refueling from 0.25% to 3%.

As for hydrogen emissions across the entire value chain, several publications proposed an estimate that ranges between 0.2% and 20% (e.g., Bond et al., 2011; Cooper et al., 2022; Warwick et al., 2022). Certain studies noted that emissions exceeding 10% typically only occur in specific scenarios, such as uncontrolled liquid hydrogen evaporation. Esquivel-Elizondo et al. (2023) highlight that the challenge in establishing a definitive range for the entire value chain arises from the differing definitions and scopes used across numerous studies.

#### Emission rate calculation

To estimate hydrogen emissions, we required emission rates per activity. Here, emission refers to hydrogen released from equipment, pipelines, and other infrastructure used in production, storage, distribution processes, and intentional emissions such as purging and/or venting. These emission rates were derived from preliminary data published in a literature review by Esquivel-Elizondo et al. (2023) and can be found in Table 2. Given the significant uncertainty in these estimates, a range was adopted for each source to reflect variability. For emissions from end-use, where data is particularly scarce, the same range of 0.2% to 0.5% reported by Fan et al. (2022) and Frazer-Nash Consultancy (2022) for end-use chemical and refining was adopted. It was applied to all end-use sectors, including methanol and ammonia, as there were no alternatives. In contrast, distinct emissions ranges were selected for production processes, including grey and blue hydrogen (SMR with and without CCS, and by-product) and green hydrogen (electrolysis). In this case, by-product hydrogen is assumed to be mostly grey hydrogen due to the nature of its production, which typically involves processes like natural gas reforming or refinery off-gases. This assumption stems from the fact that by-product hydrogen is often linked to fossil fuel-derived operations, making it a source of carbon emissions like grey hydrogen. However, this classification is not always immediately apparent, as by-product hydrogen can, in some instances, be produced through processes with lower carbon footprints. The chlor-alkali process represents such an exception. In this process, hydrogen is generated as a secondary product through the electrolysis of sodium chloride solution.^51^ Thus, the hydrogen emissions associated with chlor-alkali by-product production align more closely with those of traditional water electrolysis rather than grey hydrogen production and, as a result, the same emission rate as green hydrogen was used. For pipelines, it was assumed that hydrogen pipelines would behave similarly to natural gas transportation and storage pipelines, with the hydrogen emission rate calculated by adjusting the methane (CH_4_) emission rate based on the H_2_/CH_4_ mass ratio.

To apply these emission rates, we first needed to determine the operational capacities of various hydrogen production processes. The available data only provided total capacities, so we calculated operational capacities by first deriving the utilization rate. This rate was calculated from the ratio of annual output to annual capacity as provided by the EHO (2024a, 2024b) for each country, with an average of 73%. Notably, water electrolysis exhibited a lower utilization rate of 68%, primarily due to its reliance on intermittent renewable energy sources, which can cause fluctuations in electricity availability. Additionally, high operational costs and the variability in renewable energy supply contribute to this reduced utilization (Cloete et al., 2021). As a result, an average utilization rate per country per process was derived to determine the operating capacities, which were then multiplied by the respective emission percentage ranges to estimate hydrogen emissions.

#### Combustion emissions

##### Emissions from internal combustion engines used for road transport

To derive hydrogen emission factors for road transport combustion engines, a comprehensive literature review was conducted. The review focused especially on collecting data on H_2_/CO emission ratios. The data collection process involved a systematic search for peer-reviewed articles, reports, and studies on hydrogen emissions from road transport engines. Aalto et al. (2009) examined atmospheric hydrogen variations and traffic emissions at an urban site in Finland, reporting an H_2_/CO slope of 0.43±0.03 ppb (H_2_)/ppb (CO) during morning rush hours, which was corrected to 0.49 ppb (H_2_)/ppb (CO) after considering hydrogen soil deposition. Hammer et al. (2009) investigated H_2_/CO emission ratios from combustion sources in southwest Germany, providing corrected (between 0.45±0.003 and 0.48±0.07) and uncorrected values (between 0.45±0.003 and 0.31±0.05) for various settings, including urban rush hours and pollution events. Vogt (2009) provided insights into global hydrogen emissions and their correlation with CO emissions, highlighting significant variance in H_2_/CO ratios across different locations and conditions. Grant et al. (2010) conducted high-frequency urban measurements of hydrogen and CO in the UK, offering comparative H_2_/CO ratios for various urban settings. Vollmer et al. (2010) analyzed molecular hydrogen emissions and their isotopic signatures from motor vehicles, emphasizing differences between gasoline and diesel vehicles. Bond et al. (2010, 2011) explored hydrogen emission factors for different vehicle technologies and driving conditions, providing detailed emission data for gasoline and diesel vehicles. Additionally, Naus et al. (2018) investigated the isotopic composition of CO in vehicle exhaust and provided H_2_/CO ratios, showing a weighted average ratio of 0.71±0.31 ppb (H_2_)/ppb (CO) from individual vehicle measurements.

The synthesis of the collected data involved several steps to derive representative emission factors for hydrogen from road transport. A comparative analysis was first conducted to identify consistent patterns and outliers in the H_2_/CO emission ratios reported across multiple studies. Vollmer et al. (2010) noted that diesel vehicles produce significantly less hydrogen compared to gasoline vehicles, a finding corroborated by Bond et al. (2010) and Naus et al. (2018). Because H_2_ emissions from diesel vehicles are negligible, they were excluded from further analysis.

In the present study, we distinguished between two major vehicle categories (two-wheelers and four-wheelers) and further differentiated by Euro emission standards. While reviewing the breakdown in Bond et al. (2011), we observed that although absolute H_2_ emission factors vary considerably for older vehicles (pre-Euro 4), the H_2_/CO ratio derived from their data remains consistently close to 0.5. On this basis, we adopted a Euro class distinction between pre-Euro 4 and post-Euro 4 vehicles, also echoed by Paulot et al. (2024). For newer vehicles, higher H_2_/CO ratios were found, which Bond et al. (2011) attribute to the more stringent CO limits introduced between Euro 3 and Euro 4.^52^ These reduced CO emissions more strongly than H_2_, thereby increasing the ratio. Moreover, improvements in modern three-way catalysts compared to earlier versions, specifically in maximizing CO oxidation and promoting water–gas shift and steam reforming reactions,^53^ further support this differentiation. Two-wheelers were treated as a separate category, following the approach of Bond et al. (2010), who highlighted their significant contribution to total H_2_ emissions. For this category, we applied the H_2_/CO ratio reported specifically for two-wheelers. We did not further differentiate gasoline heavy-duty vehicles, as they represent only a marginal share of the European fleet compared to diesel vehicles and therefore have negligible influence on overall H_2_ emissions.

This robust H_2_/CO emission ratio for internal combustion engines allows for direct application to known CO emission data. For consistency with the other source sectors considered in this study (e.g., residential wood combustion and agricultural waste burning), we used the CAMS-REG-v8.1 gridded emission inventory as the basis for CO emissions from road transport. CAMS-REG-v8.1 provides a harmonized, spatially resolved emission dataset across Europe, prepared using a consistent methodology across sectors. Hydrogen emissions from gasoline-fueled road vehicles were then derived by multiplying the gridded CAMS-REG-v8.1 CO emissions with the selected H_2_/CO ratios.

##### Emissions from residential wood combustion and agricultural waste burning

Relative to road transport, hydrogen emissions from residential wood combustion remain even less studied in the scientific literature. It has, however, been observed many times that both H_2_ and CO concentrations are strongly elevated in flue gas of wood combustors. Vollmer et al. (2012) investigated hydrogen emissions from residential combustion more broadly, including two wood combustors: a small wood pellet boiler and an open fireplace, representing two extremes in combustion technology. They found that the average H_2_/CO ratios of the fireplace was about 2.5 times higher than the pellet boiler. Vollmer et al. (2012) eventually combined the results of a literature search of H_2_/CO ratios for biofuels and their own measurements, deriving an overall molar H_2_/CO ratio of 0.25 ± 0.05 for residential wood combustion, without distinguishing between appliance types.

Andreae (2019) presented an extensive compilation of biomass combustion emission factors, including average CO and H_2_ emission factors for biofuel use, from which a molar ratio of H_2_/CO ratio of 0.30 can be derived. However, the basis of the hydrogen emission factor is not entirely clear. Paulot et al. (2024) adopted Andreae (2019) value of 0.30 for their global hydrogen budget estimate. In this study, however, we have decided to follow Vollmer et al. (2012) and adopt a lower value of 0.25. This value is assumed for all types of residential wood combustion, since a robust basis for differentiating by appliance is currently lacking. Importantly, the apparent similarity between the ratios reported by Vollmer et al. (2012) and Andreae (2019)should not be interpreted as evidence of low uncertainty in our inventory results for this source.

Another biomass-related H_2_ source that is of importance on a global scale in particular, is the open burning of agricultural waste (see Paulot et al. (2024)). In Europe, however, this practice is likely less widespread, as it is legally restricted in many countries. A molar H_2_/CO ratio of 0.48 was adopted for agricultural waste burning.^54^

A spatially resolved European CO emission inventory for both residential wood combustion and agricultural waste burning is available from CAMS-REG-v8.1. Following the same approach as for road transport emissions, we prepared gridded H_2_ emissions from biomass combustion based on CAMS-REG-v8.1 CO data and the H_2_/CO ratios described above. In this study, the same H_2_/CO ratio has been applied across combustion appliance types (open fireplaces, stoves, single-house boilers, and larger boilers). We assume that appliance-specific variability is already reflected in the CAMS CO emissions. Nevertheless, further experimental research is clearly needed.

#### Gridding the datasets

To be able to compare the point, line and area sources of hydrogen emission, all sources are distributed over an equal 0.05° x 0.1° latitude – longitude grid. To create this emission grid, point and line source coordinates are rounded to the centroids of the 0.05° x 0.1° cells in which they are located. All emission sources are then summed per grid cell. The gridding process is done using the gridding tool described by Kuenen et al. (2022), that contains spatial proxies for sectors such as road transport. Plotting the cell totals of the gridded emission enables the comparison between the strengths of the various hydrogen sources. Gridded hydrogen emissions can also serve as input to chemical transport modelling.

### Quantification and statistical analysis

Quantitative analysis was conducted using Excel, and the results were reflected in Figures 1, 2, 3, 4, and 5 and Table 1. QGIS was used to display the results in Figures 2, 3, 4, and 5.

## Supplemental information

Document S1. Figures S1–S7 and Tables S1–S5

Data S1. Plant-by-plant hydrogen emissions data from the value chain, related to Figures 2 and 3
