# Supplementary material for: A gridded inventory of anthropogenic hydrogen emissions in Europe
Source: iScience. 2025 Nov 17;28(12):114095. doi: 10.1016/j.isci.2025.114095 (PMC12718198; doi:10.1016/j.isci.2025.114095)
Supplement: Document S1. Figures S1–S7 and Tables S1–S5 [file mmc1.pdf]

iScience, Volume *28*

Supplemental information

A gridded inventory of anthropogenic hydrogen emissions in Europe

Marya el Malki, Antoon Visschedijk, Ingrid Super, Jesse Duroha, Anthony J. Marchese, and Hugo Denier van der Gon

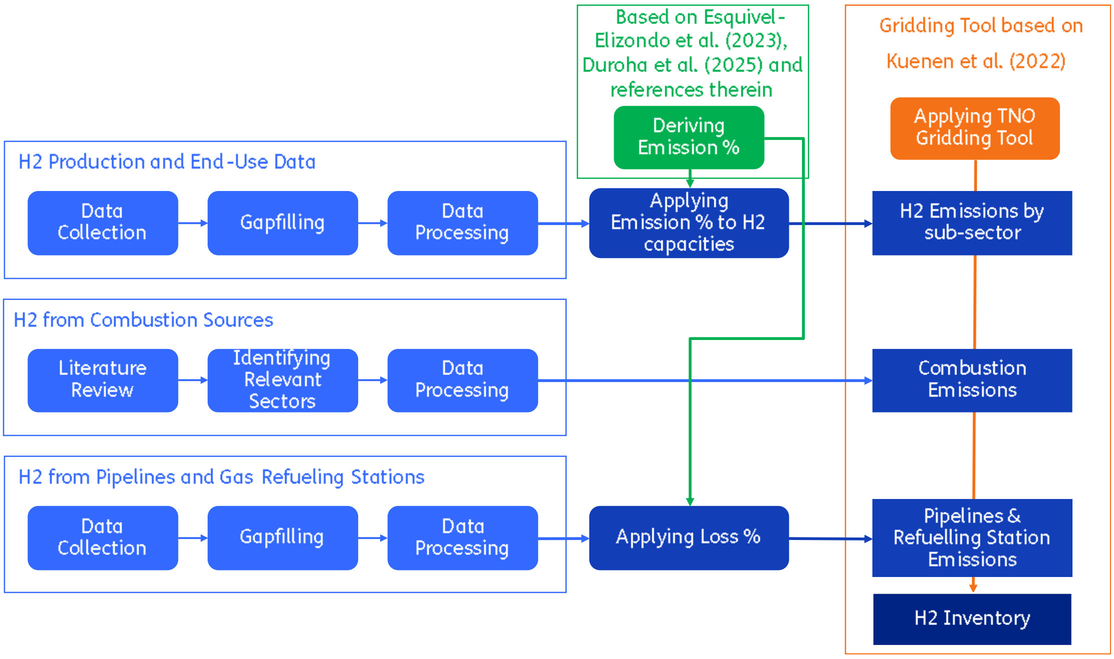

Figure S1. General methodology and categories applied to develop the hydrogen emission inventory, Related to STAR Methods.

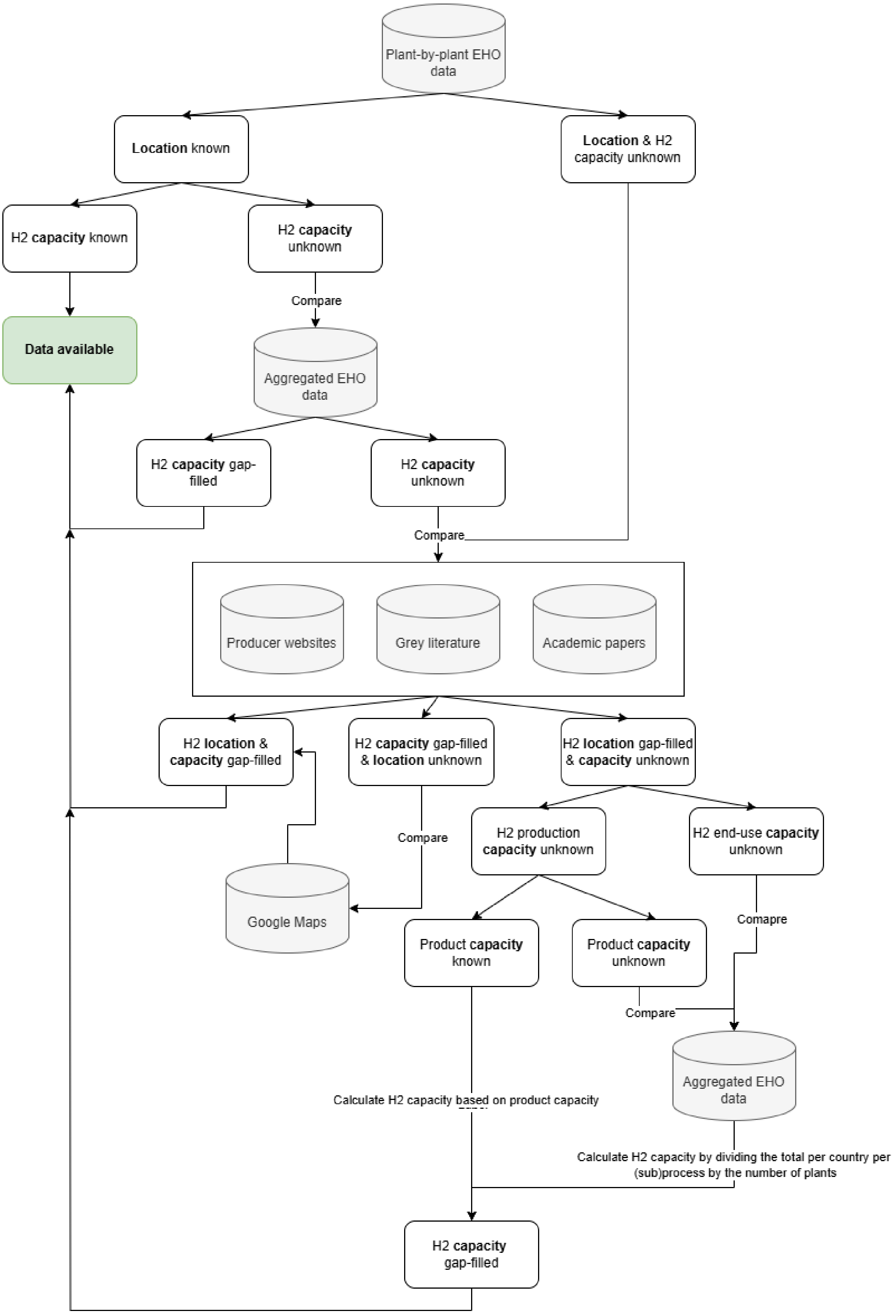

Figure S2. Gap-filling methodology for estimating the hydrogen infrastructure, Related to STAR Methods

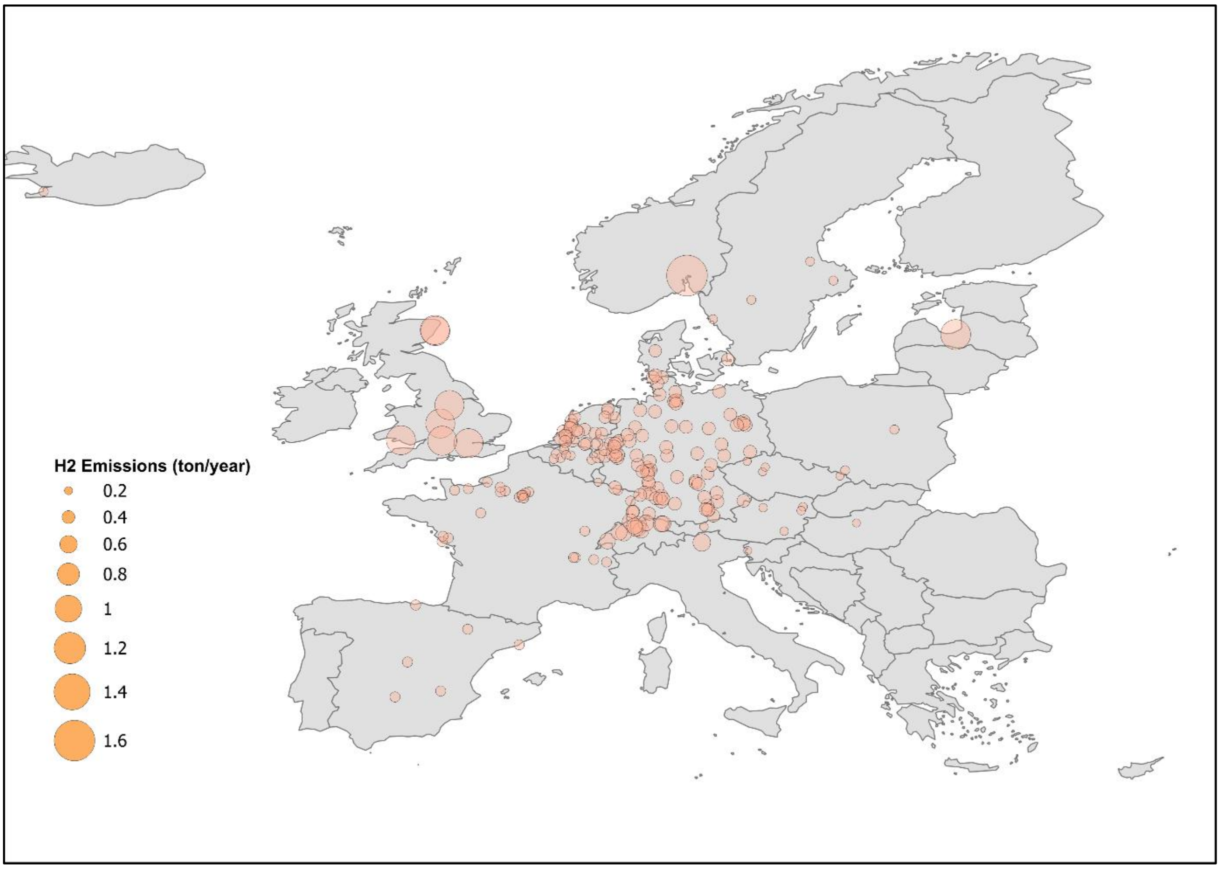

Figure S3. Hydrogen gas refueling station locations and estimated H_2 emissions across the European Domain in 2022 (EU, EFTA, and UK), Related to Figures 4 and 5.

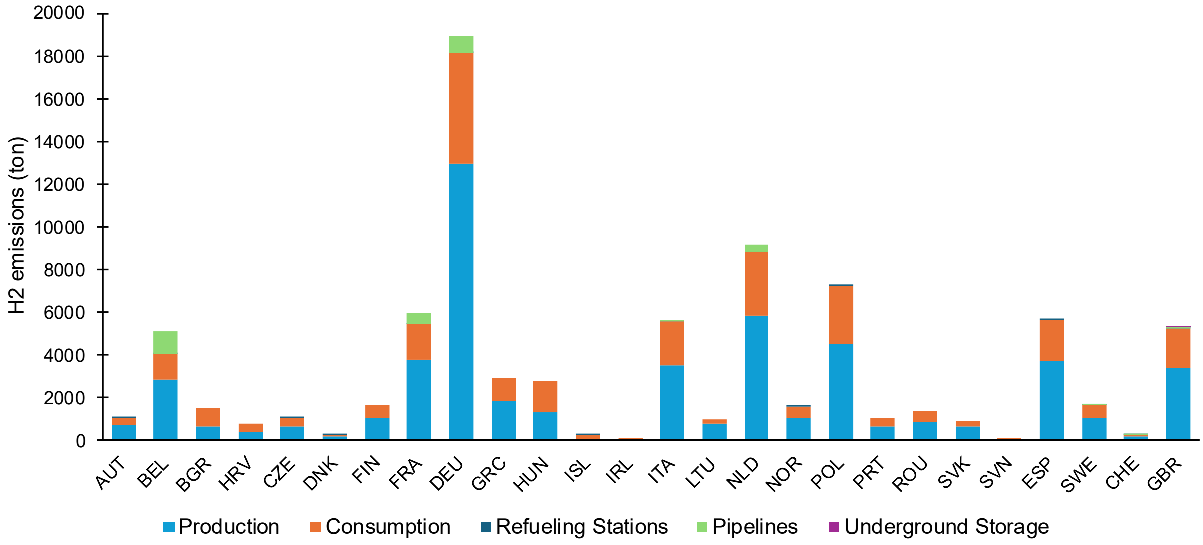

Figure S4. Average H_2 Emissions from the Hydrogen value chain, Related to Figures 4 and 5.

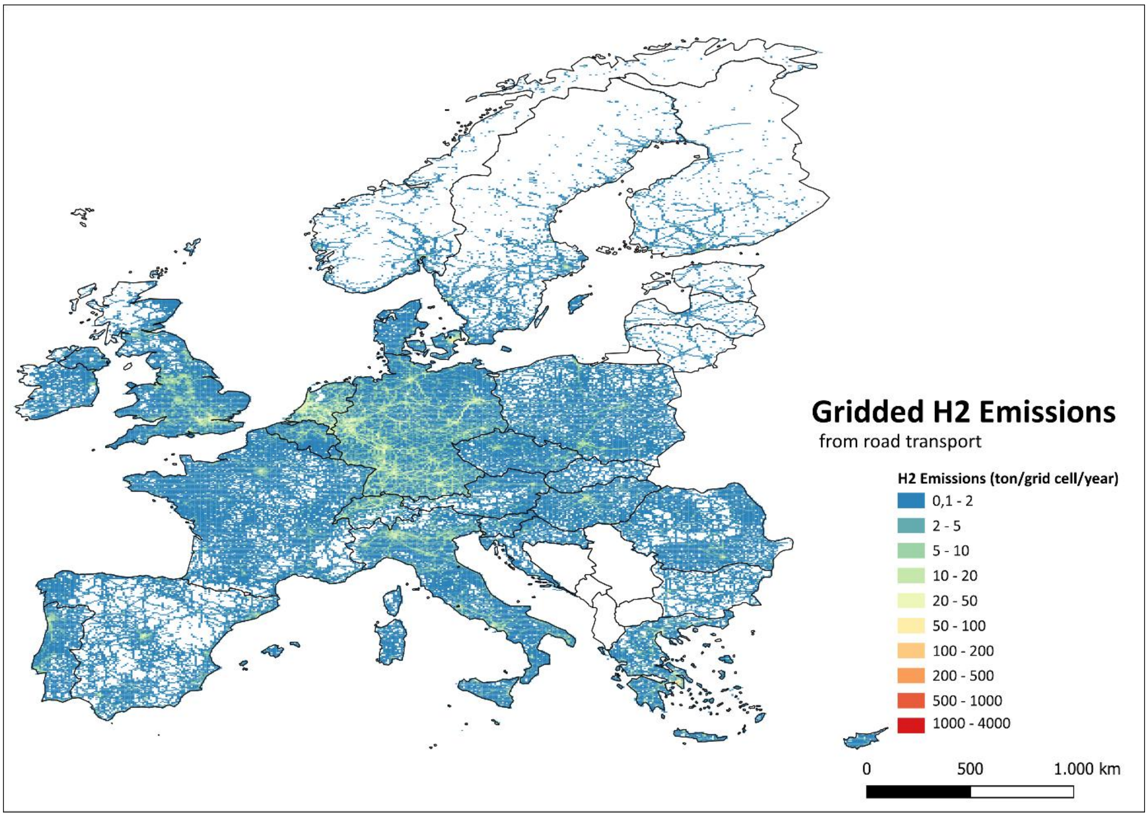

Figure S5. Hydrogen emissions (in ton/grid cell/year) from internal combustion engines as used in road transport vehicles in 2022 with a resolution of 0.05° x 0.1°, Related to Figure 5.

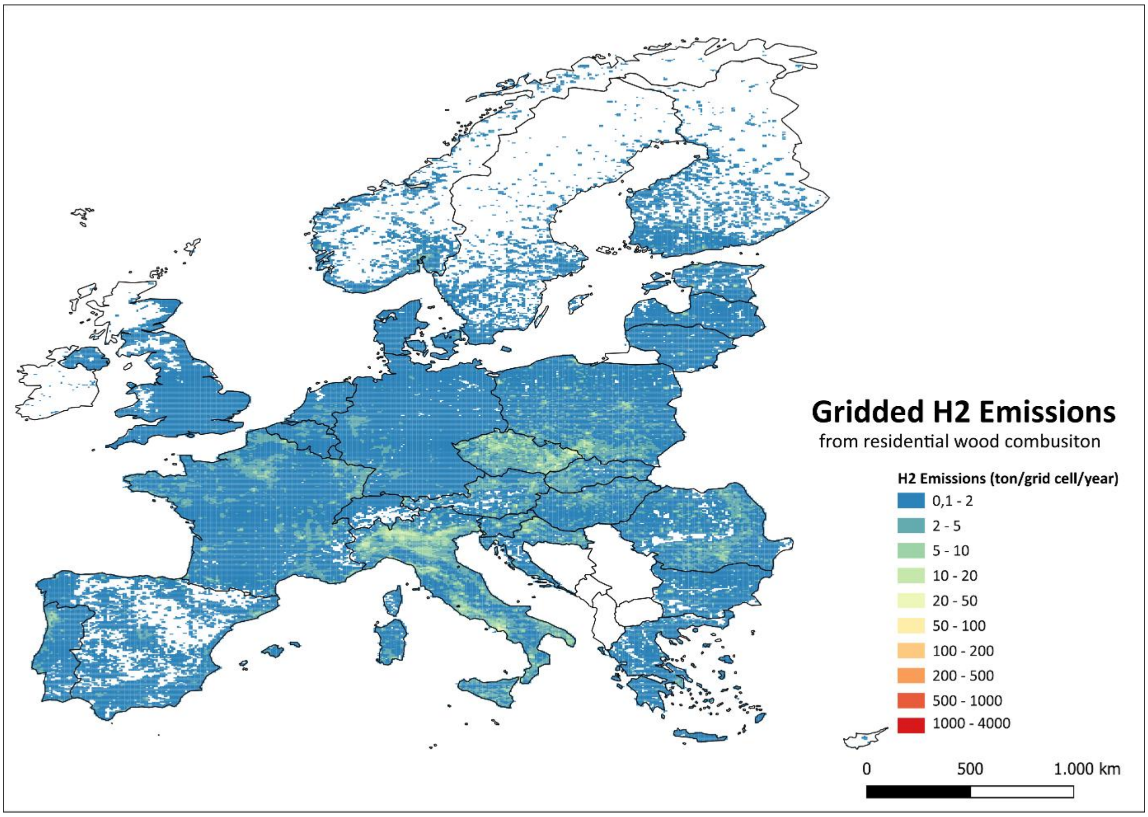

Figure S6. Hydrogen emissions (in ton/grid cell/year) from residential wood combustion in 2022 with a resolution of 0.05° x 0.1°, Related to Figure 5.

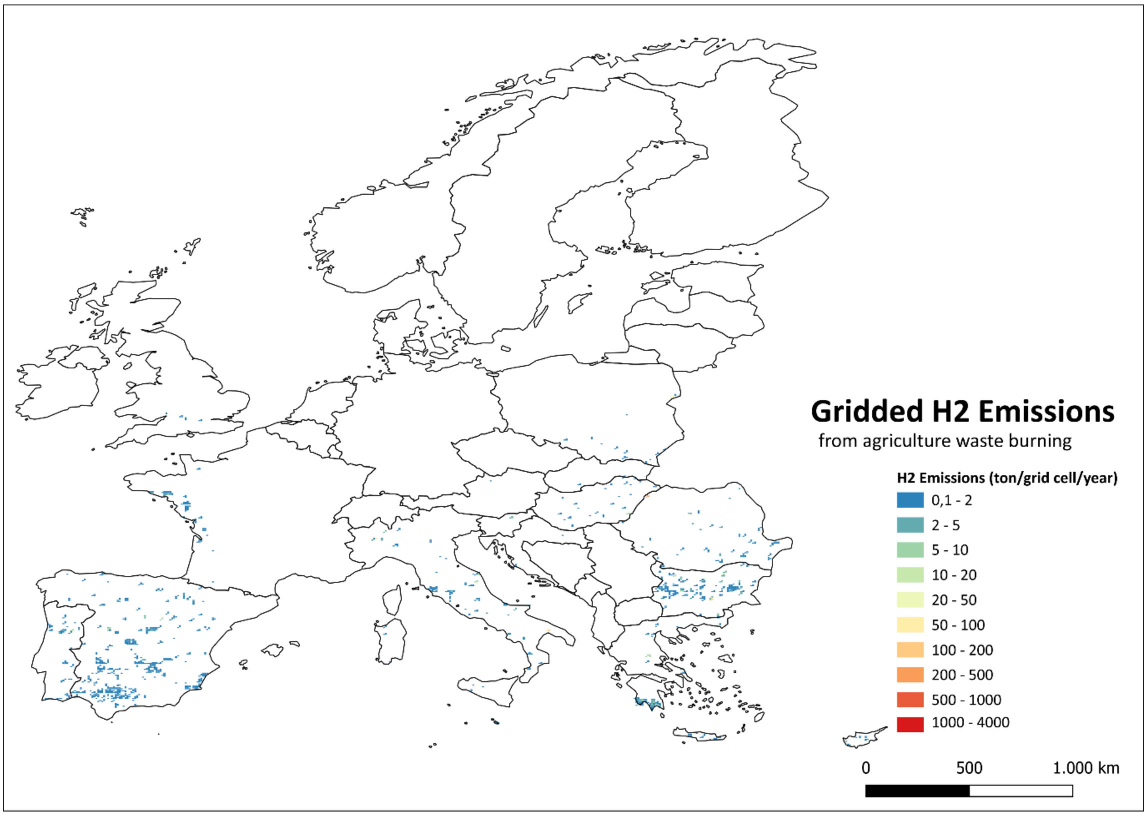

Figure S7. Hydrogen emissions (in ton/grid cell/year) from agricultural waste burning in 2022 with a resolution of 0.05° x 0.1°, Related to Figure 5.

Table S1. Absolute values of the estimated hydrogen emissions in 2022 from different sources in the hydrogen value chain and from the combustion of fuels, Related to Figure 1.

**Process**	**Hydrogen Emissions (ton/year) ***
**Production**	52300
• Electrolysis	570
• SMR	41200
• SMR with Carbon Capture	82
• By-Product	10500
By-Product Production	10500
• *Chloro-alkali*	7970
• *Ethylene*	1700
• *Sodium Chlorate*	230
• *Styrene*	560
**Combustion of fuels**	**244100**
• Road transport	125800
• Residential wood combustion	115000
• Agricultural waste burning	3300
**Pipelines**	2800
**Refueling Stations**	48
**Underground Storage**	2
**End-Use**	27700
• Ammonia	8500
• Methanol	1100
• Other Chemicals	2500
• Refining	15600
**Total**	**327000**

* For clarity, all numbers in this table (including totals) are rounded to the nearest ten for three-digit values and to the nearest hundred for larger values.

Table S2. Hydrogen emissions from production (in ton/year) for 2022 aggregated by country, Related to Figure 2.

	H2 Emissions from Production (ton/year)											
	**By-Product**			**Electrolysis**			**Reforming**			**Reforming (carbon capture)**		
**Country**	*Mean*	*Min*	*Max*	*Mean*	*Min*	*Max*	*Mean*	*Min*	*Max*	*Mean*	*Min*	*Max*
Austria	72	32	113	24	16	32	590	107	1072	0	0	0
Belgium	890	495	1284	3	2	4	1933	351	3514	0	0	0
Bulgaria	30	5	55	0	0	0	636	116	1157	0	0	0
Croatia	0	0	0	0	0	0	343	62	623	0	0	0
Czechia	107	45	168	0	0	0	508	92	923	0	0	0
Denmark	0	0	0	13	9	18	134	24	244	0	0	0
Finland	146	51	240	33	22	44	860	156	1565	0	0	0
France	1037	567	1507	20	13	27	2633	479	4788	54	10	97
Germany	4357	2689	6025	216	144	288	8387	1525	15248	0	0	0
Greece	6	4	8	3	2	4	1794	326	3263	0	0	0
Hungary	340	205	476	0	0	0	935	170	1699	0	0	0
Iceland	0	0	0	23	15	30	0	0	0	0	0	0
Ireland	6	4	9	0	0	0	42	8	77	0	0	0
Italy	337	161	514	9	6	12	3167	576	5758	5	1	9
Lithuania	0	0	0	0	0	0	790	144	1437	0	0	0
Netherlands	1215	507	1923	14	10	19	4604	837	8371	24	4	43
Norway	248	147	349	28	18	37	785	143	1427	0	0	0
Poland	304	179	430	0	0	0	4219	767	7671	0	0	0
Portugal	134	72	197	0	0	0	530	96	965	0	0	0
Romania	117	78	156	0	0	0	717	130	1304	0	0	0
Slovakia	65	35	96	0	0	0	555	101	1008	0	0	0
Slovenia	9	6	12	0	0	0	8	2	15	0	0	0
Spain	430	210	651	84	56	112	3155	574	5736	0	0	0
Sweden	250	85	416	31	21	41	774	141	1407	0	0	0
Switzerland	27	18	36	46	30	61	99	18	181	0	0	0
United Kingdom	352	176	528	21	14	28	2963	539	5386	0	0	0
**Total**	10481	5770	15191	569	379	759	41162	7484	74841	82	15	150

Table S3. Hydrogen emissions from by-product production (in ton/year) for 2022 aggregated by country, Related to Figure 2.

	**H2 Emissions from By-Product Production (ton/year)**											
	**Chlor-alkali**			**Ethylene**			**Sodium Chlorate**			**Styrene**		
**Country**	*Mean*	*Min*	*Max*	*Mean*	*Min*	*Max*	*Mean*	*Min*	*Max*	*Mean*	*Min*	*Max*
Austria	38	25	51	34	6	62	0	0	0	0	0	0
Belgium	688	459	917	160	29	292	0	0	0	41	8	75
Bulgaria	0	0	0	30	5	55	0	0	0	0	0	0
Croatia	0	0	0	0	0	0	0	0	0	0	0	0
Czechia	53	35	70	43	8	78	0	0	0	11	2	21
Denmark	0	0	0	0	0	0	0	0	0	0	0	0
Finland	51	34	68	32	6	58	62	11	114	0	0	0
France	780	520	1040	177	32	321	30	5	55	50	9	91
Germany	3913	2609	5217	377	69	686	0	0	0	67	12	122
Greece	6	4	8	0	0	0	0	0	0	0	0	0
Hungary	295	197	393	45	8	83	0	0	0	0	0	0
Iceland	0	0	0	0	0	0	0	0	0	0	0	0
Ireland	6	4	9	0	0	0	0	0	0	0	0	0
Italy	205	137	273	83	15	150	3	0	5	47	9	85
Lithuania	0	0	0	0	0	0	0	0	0	0	0	0
Netherlands	590	394	787	320	58	582	0	0	0	305	55	554
Norway	209	140	279	38	7	70	0	0	0	0	0	0
Poland	254	169	339	50	9	91	0	0	0	0	0	0
Portugal	98	66	131	32	6	58	4	1	8	0	0	0
Romania	117	78	156	0	0	0	0	0	0	0	0	0
Slovakia	48	32	64	17	3	31	0	0	0	0	0	0
Slovenia	9	6	12	0	0	0	0	0	0	0	0	0
Spain	271	181	361	110	20	199	11	2	20	39	7	70
Sweden	81	54	108	46	8	83	124	22	225	0	0	0
Switzerland	27	18	36	0	0	0	0	0	0	0	0	0
United Kingdom	231	154	308	121	22	220	0	0	0	0	0	0
**Total**	7971	5314	10628	1715	312	3118	234	43	426	560	102	1019

Table S4. Hydrogen emissions from refueling stations (in ton/year) for 2022 aggregated by country, Related to Figures 4, 5 and S3.

**Country**	**Sum of Total # of dispensers**	**Average of Average year demand (ton)**	**H2 Emissions from Refueling Stations (ton/year)**		
			Mean	*Min*	*Max*
Austria	5	1.61	0.13	0.02	0.24
Belgium	11	3.38	0.44	0.07	0.81
Czechia	6	0.56	0.04	0.01	0.07
Denmark	4	13.02	0.42	0.07	0.78
France	56	5.62	2.28	0.35	4.22
Germany	113	14.69	20.54	3.16	37.91
Hungary	2	0	0.00	0.00	0.00
Iceland	1	3.36	0.05	0.01	0.10
Italy	3	28.32	0.46	0.07	0.85
Latvia	2	66	1.07	0.17	1.98
Luxembourg	3	0.2	0.00	0.00	0.01
Netherlands	55	12.17	5.14	0.79	9.49
Norway	2	297.42	4.83	0.74	8.92
Poland	6	3.32	0.11	0.02	0.20
Slovenia	2	0	0.00	0.00	0.00
Spain	9	6.24	0.61	0.09	1.12
Sweden	6	2.92	0.19	0.03	0.35
Switzerland	21	23.70	4.62	0.71	8.54
United Kingdom	16	63.57	7.23	1.11	13.35
**Total**	323	546	48	7	89

Table S5. Hydrogen emissions from end-use (in ton/year) for 2022 aggregated by country, Related to Figure 3.

	**H2 Emissions from End-Use (ton/year)**											
	**Ammonia**			**Methanol**			**Other chemicals**			**Refining**		
**Country**	*Mean*	*Min*	*Max*	*Mean*	*Min*	*Max*	*Mean*	*Min*	*Max*	*Mean*	*Min*	*Max*
Austria	234	134	334	0	0	0	14	8	20	126	72	181
Belgium	476	272	680	0	0	0	149	85	213	589	337	841
Bulgaria	589	337	841	0	0	0	0	0	0	242	139	346
Croatia	242	139	346	0	0	0	0	0	0	184	105	263
Czechia	184	105	263	0	0	0	54	31	76	163	93	233
Denmark	0	0	0	0	0	0	0	0	0	85	49	122
Estonia	0	0	0	0	0	0	0	0	0	0	0	0
Finland	0	0	0	0	0	0	103	59	147	497	284	710
France	531	303	759	0	0	0	102	58	146	1073	613	1533
Germany	1253	716	1791	528	302	755	346	198	495	3055	1746	4364
Greece	1	1	2	0	0	0	0	0	0	1067	610	1524
Hungary	1067	610	1524	0	0	0	232	132	331	194	111	277
Iceland	0	0	0	194	111	277	0	0	0	0	0	0
Ireland	0	0	0	0	0	0	1	0	1	27	15	38
Italy	272	156	389	0	0	0	39	23	56	1753	1002	2504
Lithuania	0	0	0	37	21	53	0	0	0	165	94	236
Netherlands	915	523	1307	73	41	104	790	452	1129	1163	664	1661
Norway	227	130	324	149	85	212	7	4	10	130	74	185
Poland	1310	749	1872	29	16	41	48	28	69	1333	762	1905
Portugal	0	0	0	0	0	0	34	19	48	307	175	438
Romania	307	175	438	12	7	17	0	0	0	249	143	356
Slovakia	249	143	356	0	0	0	6	3	8	1	1	2
Slovenia	0	0	0	0	0	0	5	3	8	0	0	0
Spain	232	133	331	0	0	0	62	35	89	1676	958	2395
Sweden	0	0	0	50	29	72	83	48	119	440	252	629
Switzerland	0	0	0	0	0	0	24	14	35	43	25	62
United Kingdom	414	237	592	0	0	0	403	230	576	1081	618	1545
**Total**	8505	4860	12151	1071	612	1531	2503	1430	3576	15644	8939	22349
